# Incidental Discovery of a Cervical Rib in a Case of Acute Cholecystitis: A Rare Anatomical Variant

**DOI:** 10.7759/cureus.58794

**Published:** 2024-04-22

**Authors:** Pratik S Navandhar, Pankaj Gharde, Raju K Shinde, Tushar Nagtode, Bhagyesh Sapkale

**Affiliations:** 1 General Surgery, Jawaharlal Nehru Medical College, Datta Meghe Institute of Higher Education and Research, Wardha, IND; 2 Medicine, Jawaharlal Nehru Medical College, Datta Meghe Institute of Higher Education and Research, Wardha, IND

**Keywords:** radiographic examination, anatomical variant, incidental discovery, acute cholecystitis, cervical rib

## Abstract

This case report discusses the incidental discovery of a cervical rib in a 53-year-old woman presenting with acute cholecystitis. While cervical ribs are rare, their identification holds clinical significance due to their potential implications for vascular compression or thoracic outlet syndrome. Despite the patient's primary complaint of cholecystitis, a chest X-ray incidentally revealed the cervical rib. This finding underscores the importance of a thorough radiographic examination. The report discusses similar cases, emphasizing varying clinical presentations and associated vascular complications. The case highlights the necessity for a comprehensive assessment of incidental findings to ensure holistic patient care and management, emphasizing the importance of considering anatomical variants in clinical practice.

## Introduction

A congenital overdevelopment of the transverse process of a cervical spine vertebra is called a cervical rib, sometimes referred to as a neck rib or a supernumerary rib in the cervical region [1]. The seventh cervical vertebra gives rise to these anatomically rare ribs, which affect 0.5-1% of people [1]. It is present usually without any symptoms, but it may lead to vascular compression or thoracic outlet syndrome (TOS) [2,3]. Children with cervical ribs frequently exhibit no symptoms other than a neck lump and slight soreness [2]. Cervical ribs can be unilateral or bilateral and differ in size, shape, and attachment sites [3]. The majority of cervical ribs are clinically insignificant and go unnoticed throughout life. They may, however, occasionally result in localized discomfort and compress nearby structures, necessitating medical attention.

Usually, the cervical rib comprises a head, neck, and tubercle [1]. A fibrous band close to the anterior scalene muscle's insertion attaches it posteriorly to the first rib. For cervical ribs to be classified as ribs, they must articulate with the transverse process [1,4]. The left side of the body is more likely than the right to have unilateral cervical ribs [4]. Compared to bilateral cervical ribs, unilateral cervical ribs are more common [2,5].

Cervical ribs, variations of rib-like structures originating from the cervical vertebrae, are categorized into four types based on their distinct characteristics. Type 1 cervical ribs are fully formed ribs that typically connect to either the first rib or the manubrium, a part of the sternum [1,5]. Type 2 cervical ribs are incomplete, lacking a full connection, and often have a free distal tip [3]. Type 3 cervical ribs also lack a complete connection and are characterized by a distal attachment via a fibrous band [1,4]. Type 4 cervical ribs are relatively short bone segments that extend beyond the transverse process of the C7 vertebra [1,3,4]. Despite their variations, cervical ribs do not serve any known physiological function in the body. In this case report, a cervical rib was unintentionally found during radiographical imaging on a 53-year-old lady who had cholecystitis.

## Case presentation

A 53-year-old female patient was presented at Acharya Vinoba Bhave Rural Hospital (AVBRH) with symptoms of nausea, vomiting, and stomach ache in the upper right quadrant. Our hospital's clinical examination found optimistic Murphy's sign and tenderness. Laboratory investigation results showed leukocytosis and raised liver enzymes. Abdominal ultrasound depicted acute cholecystitis.

In one particular situation, a chest X-ray was performed accidentally, as it happened to be just one of the patient's symptoms that were being investigated for acute cholecystitis. Although the main complaint had been acute cholecystitis, some healthcare providers would sometimes order other imaging studies to exclude other probable issues, or an unexpected result from an X-ray might be curious enough for additional diagnostic workup.

**Figure 1:**
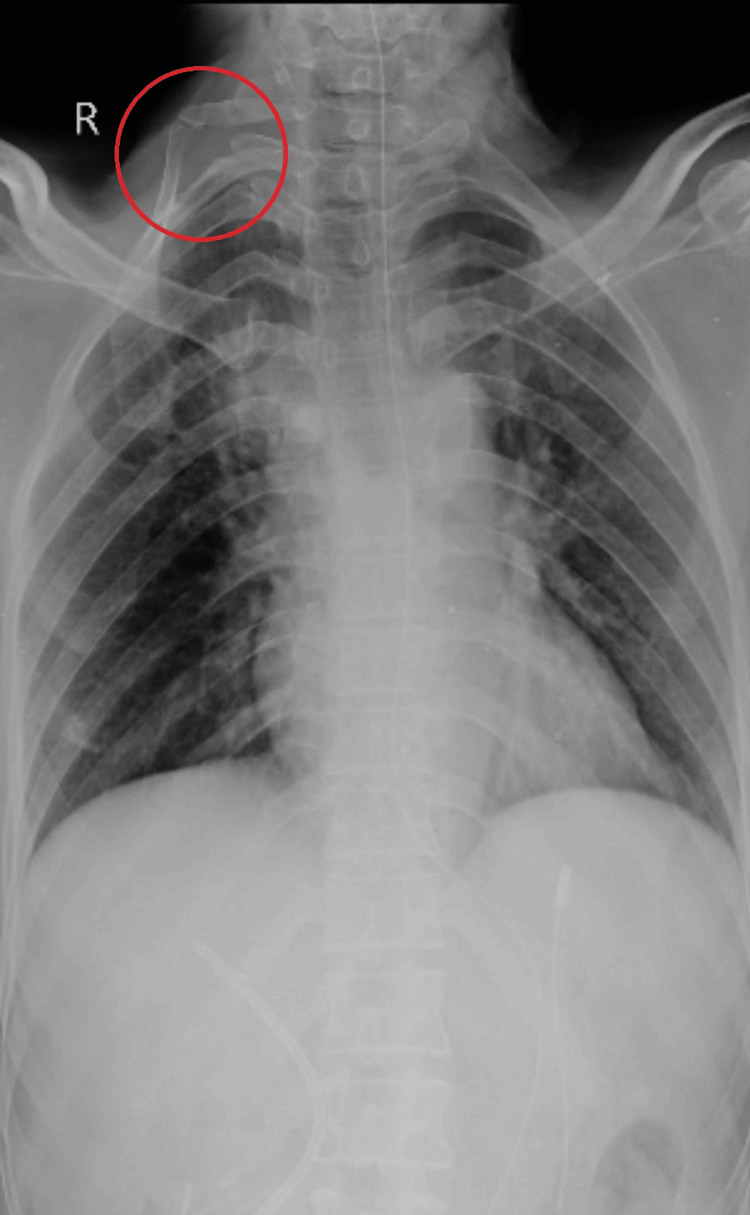
The right side of the cervical region showing the cervical rib

Furthermore, detecting the cervical rib may help plan future medical procedures or assess any likely consequences it may impose on the patient's well-being. Thus, the incidental finding of the cervical rib on a chest X-ray supplements the workup despite not being the reason for admission. The timeline of events in this care is highlighted in Table 1, whereas Figure 1 depicts the cervical rib on the right side of the cervical region.

**Table 1:** Timeline of events

Year	Event
Day 1	The patient presented with abdominal pain
Day 1	Positive Murphy's sign observed
Day 2	Abdominal ultrasound confirms cholecystitis
Day 2	Incidental finding of cervical rib on chest X-ray

## Discussion

The observance of the type 1 cervical rib on the chest X-ray was an incidental finding in this case of a 53-year-old female who was presented at AVBRH for symptoms like right upper quadrant abdominal pain, nausea, and vomiting. Extra cervical ribs grow above the first rib, often originating from the seventh cervical vertebra [4].

Yadav et al. provided a series of cases from a Nepalese University Hospital that clarifies cervical ribs' therapy and clinical presentation, mainly concentrating on a younger group with a 20-year average age [5]. Interestingly, the acute stomach symptoms our patient experienced are not the same as those listed in their series, which include neck discomfort, mass in the neck, tingling sensation, and weakness on the afflicted side. It's important to realize, though, that cervical ribs can cause a wide range of symptoms, or none at all in many situations, as this 53-year-old woman's does.

A 32-year-old woman complained of pain, coldness, and swelling in her left upper limb in a case reported by Lokanayaki, which was suggestive of vascular compromise [6]. The presence of bilateral cervical ribs was confirmed by imaging examinations, which resulted in compression of the left subclavian artery, stenosis, and the creation of a post-stenotic aneurysm. The vascular consequences of cervical ribs are highlighted in this case, as they are linked to TOS and acute limb artery blockage [7]. Diagnosis of TOS can be challenging and often requires a thorough clinical examination. Symptoms can vary widely among individuals but commonly include shoulder and arm pain, weakness, and paresthesia (tingling or numbness). A clinical evaluation usually includes determining whether the affected arm has any soreness, muscle weakness, altered feeling, or irregular pulse strength or rhythm [6,7]. To further assess the structure of the thoracic outlet and identify any potential compression of blood vessels or nerves contributing to the symptoms, diagnostic imaging such as MRIs, ultrasounds, or X-rays may be used in addition to a clinical examination [7].

In a case, a seven-year-old girl presented with a longstanding swelling on the right side of her neck, which was discovered since birth [8]. Although the swelling was painless, the mother expressed concerns about potential complications. Upon examination, a 4 cm × 4 cm hard and non-tender swelling was palpable over the right supraclavicular region. A chest X-ray incidentally revealed bilateral cervical ribs [8]. Subsequent X-ray imaging of the soft tissue neck confirmed the presence of bilateral cervical ribs, with complete formation on the right side and incomplete on the left [8]. This case is suggestive that the cervical rib can be symptomless in many cases, as in our case of a 53-year-old female patient presented at AVBRH.

A 50-year-old South African man's case sheds light on a rare anatomical variation involving a unilateral, right-sided cervical rib synostosis to the first thoracic rib, which the authors have termed a cervicothoracic rib complex [9]. The morphological features observed in this case include increased angulation, widening of the body, and shortening of the right-sided first thoracic rib, with synostosis occurring at the angle of the first thoracic rib. Additionally, the cervical rib exhibited a well-defined head with an articular facet, followed by a short neck and a similar-sized articulating facet at the tubercle. Radiographic analysis revealed alterations in the trabecular bone pattern consistent with the gross osteologic appearance described, along with an elliptical lucent zone representing the fusion site to the first thoracic rib [9]. This case underscores the importance of detailed radiological assessment and a thorough understanding of anatomical variations, particularly in the context of potential surgical interventions, as also highlighted in our case of a 53-year-old female.

## Conclusions

In conclusion, the incidental discovery of a cervical rib in a patient presenting with acute cholecystitis underscores the importance of a thorough radiographic examination in clinical practice. While cervical ribs are rare anatomical variants, their identification can have implications for future medical procedures and may prompt further investigation into associated conditions such as TOS. The recognition of TOS requires diligent clinical examination, including assessment for symptoms such as shoulder and arm pain, weakness, and paresthesia. This case highlights the need for comprehensive assessment and consideration of incidental findings to ensure holistic patient care and management.
